# The characteristics of adjacent anatomy of mandibular third molar germs: a CBCT study to assess the risk of extraction

**DOI:** 10.1038/s41598-017-14144-y

**Published:** 2017-10-26

**Authors:** Rui Sun, Yu Cai, Yi Yuan, Ji-Hong Zhao

**Affiliations:** 1 The State Key Laboratory Breeding Base of Basic Science of Stomatology (Hubei-MOST) & Key Laboratory of Oral Biomedical Engineering of Ministry of Education, School & Hospital of Stomatology, Wuhan University, Wuhan, P.R. China; 2 Department of Oral and Maxillofacial Surgery, School and Hospital of Stomatology, Wuhan University, Wuhan, P.R. China; 3 Department of Oral Radiology, School and Hospital of Stomatology, Wuhan University, Wuhan, P.R. China

## Abstract

This study aims to investigate the characteristics of adjacent anatomy of mandibular third molar germs (MTMGs). Three hundred Chinese patients aged 12 to 17 years old who received cone-beam computed tomography (CBCT) were enrolled. The continuity of cortical outline of inferior alveolar canals (IACs) in the region of MTMGs, the integrity of lingual bone cortex and the relationship between hard tissue part of MTMGs and IACs were investigated by observing CBCT data via the NNT viewer software. The age, degree of dental development, gender and location were recorded as variables. The associations between different variables and the observed data were analysed. The possibilities of disrupted cortical outline of IACs or the hard tissue part of MTMGs contacting IACs were significantly lower in 12 or 13 age groups, lower in Nolla stage ≤ 6. Males were significantly less than females in the incidence of disrupted cortical outline of IACs. As to the perforation of lingual bone cortex, no significant differences were observed in gender, ages, location or development stages. According to the CBCT images, anatomical factors contributed the lest to the risk of inferior alveolar nerve and lingual nerve injury in the 12 to 13 age group during removing the MTMG removal.

## Introduction

Impacted mandibular third molars are associated with many kinds of complications and disorders, including dental crowding^1^, pericoronitis, tooth caries^2^, root resorption^3^ and periodontal problems^4^. Serious complications, such as severe inflammatory responses or even life-threatening, osteomyelitis of the mandible^5^ and development of cysts and tumours^6^, require hospitalization for treatments. During the third molar eruption, impaction would occur by some reasons; among which, lacking of the bone space may be the most significant factor^7^.

To avoid the crowded dentition, prophylactic removal of the mandibular third molar germs (MTMGs) should be widely performed, which is suggested by orthodontists^8^. However, MTMGs were totally embedded by the bone or soft tissues. Moreover, bone or soft tissue resistance should be relieved before the extraction. Therefore, the relationships between important adjacent anatomical structure and tooth germs should be assessed. However, no summary was available to describe the characteristics of adjacent anatomy around the MTMGs. In the present study, the anatomical characteristics of 300 MTMGs, obtained from 12- to 17-year-old patients were scanned by cone-beam computed tomography (CBCT) and measured by NNT viewer software.

## Materials and Methods

### Study design and sample

Chinese patients aged 12 to 17 years old, who received a CBCT in the Department of Radiology, Hospital of Stomatology Wuhan University were enrolled in our study. This study followed the tenets of the Declaration of Helsinki for research involving human subjects. Informed consents were obtained from all legal guardians of the participants. The study was critically reviewed and approved by the institutional review board of Hospital of Stomatology Wuhan University (Wuhan, China).

Exclusion criteria were as follows: 1. patients with no MTMGs; 2. the MTMGs have associated cystic lesions; 3. patient with dentoalveolar deformities; 4. the adjacent mandibular second molars were missing; and 5. those who suffered from mandible fracture.

Enrolling procedure includes the following. Data of patients who received CBCT before December 31, 2016 were collected in a reversed order according to the recording date. After filtering, data were chosen successively until the number of cases in each mental age group reached up to 50 (total of six age groups, 12–17 age groups). Altogether, 300 MTMGs were enrolled in our study. Specifically, 151 tooth germs were in the left side of mandible and 149 were in the right side. The male/female ratio was 1:1.13 (Table 1).

**Table 1:** Basic information about the teeth germs.

Group	Age	Male	Female	Left	Right
A	12~13	40	60	50	50
B	14~15	50	50	50	50
C	16~17	52	48	51	49
	Total	142	158	151	149

### CBCT imaging acquisition

The images of all patients were scanned by the same machine NewTom VG (Quantitative Radiology, Verona, Italy) and manipulated by one radiologist (Yuan Yi). The scanner specifications are listed as follows: focal spot 0.3 mm, scan time of 18 s to 26 s, X-ray emission time of 3.6 s, axial thickness of 0.300 mm, axial pitch of 0.300 mm and field of view of amorphous silicon flat panel of 15 cm × 15 cm. During scanning, the Frankfort horizontal planes of patients were paralleled to the ground. The facial midlines were also coherent with the long axis of the machine.

### Measurement procedure and data classification

**Figure 1:**
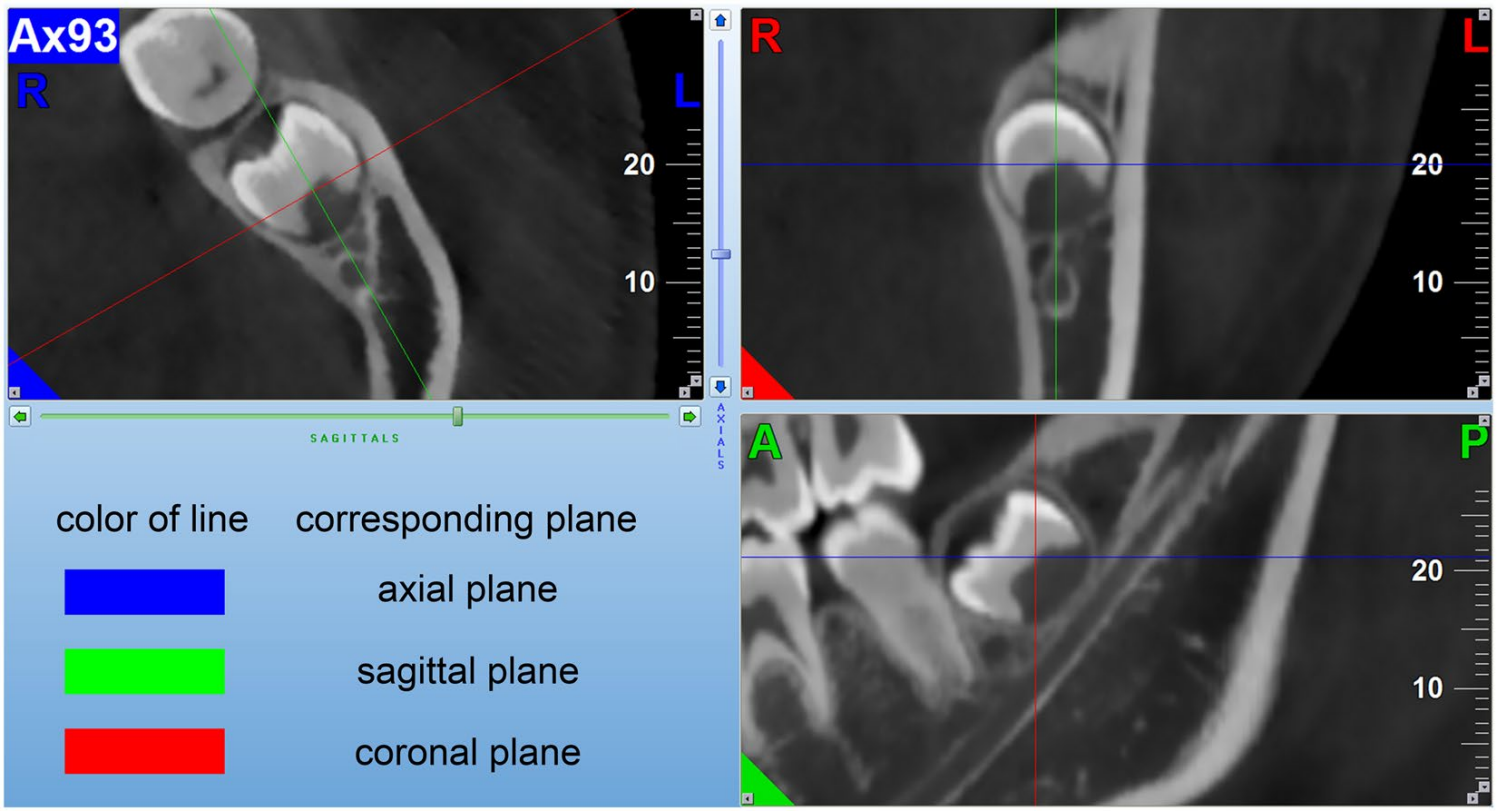
The interface of NNT Viewer software program (MPR pattern) was showed, and three screens respectively represent axial image, coronal image and sagittal image. This figure also indicated the methods to investigate the relationship between IACs and MTMGs.

NNT Viewer software program was used to analyse the CBCT image. The pattern of Serial multiple reformatted image (MPR) in the software was used for image observation. Three screens were available representing axial image, coronal image and sagittal image in MPR. In each screen, two vertical intersecting lines which referred to the other two screens can be controlled. Before our observation, the sagittal image was adjusted to the direction parallel to the long axis of IAC in the MTMG region. (Fig. 1). The integrity of the lingual cortex and the relationship between the inferior alveolar canals (IACs) and MTMGs were recorded.

The methods to investigate the relationship between IACs and MTMGs were described as follows. Given the similar radiology manifestation between the IAC and the cancellous bone of the mandible, the IAC was first identified through the mandibular foramen in axial screen. Then, the axial images were altered to investigate the continuity of the cortical outline of IAC. The sagittal and coronal images were also used to confirm the IAC continuity. After the discontinuous region of IAC was found in the axial image, the sagittal image was used to observe the relationship of the hard tissue part of MTMG and the white line border of IAC. The computed tomography image slices including proof of the discontinuity of cortical outline of IAC, the closest distance between IAC and the MTMGs and the relationship of the hard tissue part of MTMGs and the white line border of IACs were screen captured (Fig. 1).

**Figure 2:**
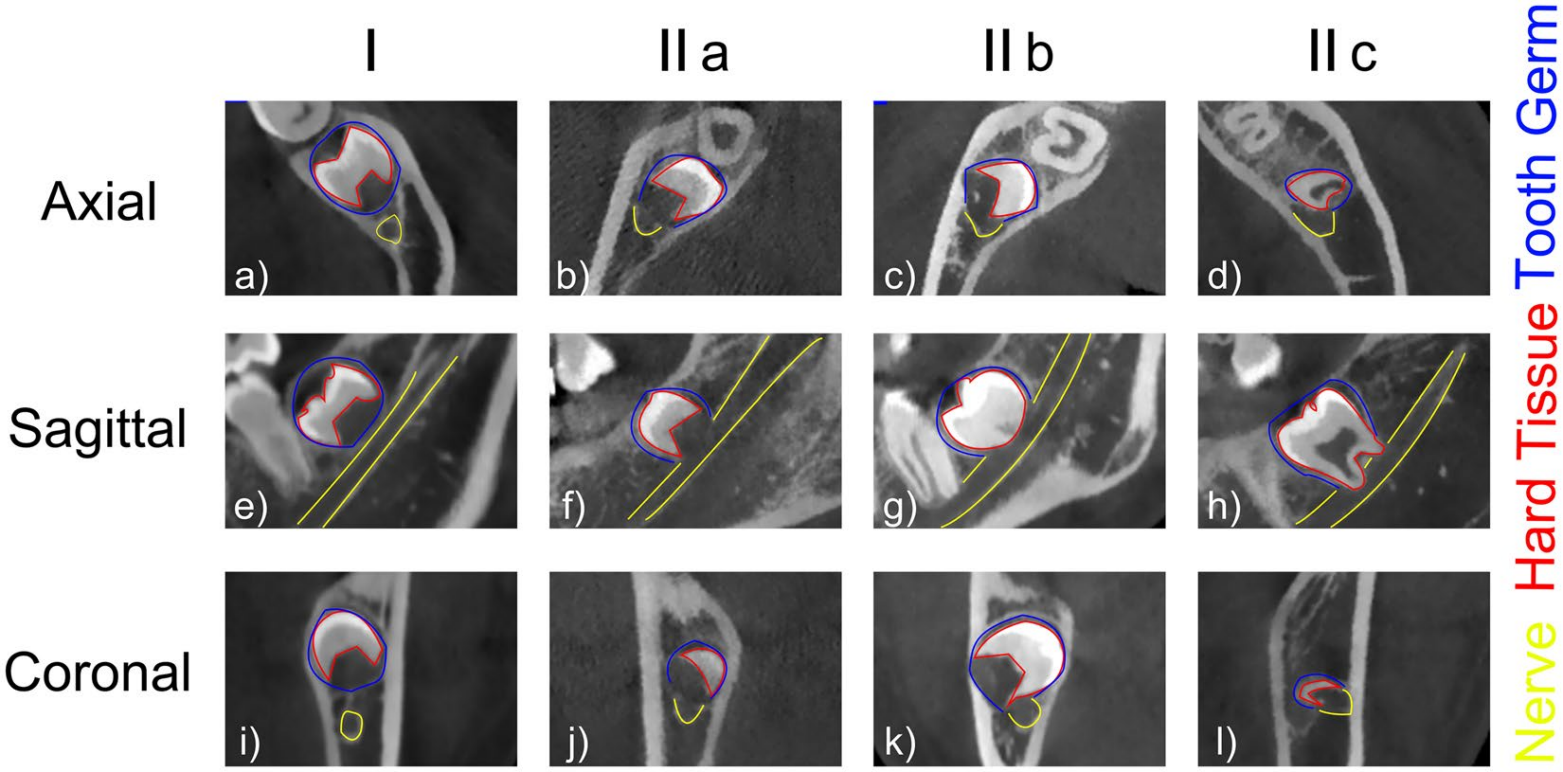
Relationship between the MTMGs and IACs in axial, sagittal, coronal planes. Yellow line indicates outline of nerve, red line means outline of hard tissue part of MTMG, blue line shows outline of MTMG. (**a**–**d**) Axial images; (**e**–**h**) sagittal images; (**i**–**l**) coronal images. (**a**,**e** and **i**) Images of level I, (**b**,**f** and **j**) images of IIa, (**c**,**g** and **k**) images of IIb, (**d**,**h** and **l**) images of IIc.

The classification of the relationship between IACs and MTMGs followed the category described by Ghaeminia^9^ with minor modifications. The relationship between IACs and MTMGs was classified into two levels first based on the continuity of the cortical outline of IACs in the region of MTMGs: (level I) continuous cortical outline (Fig. 2a,e and i); (level II) disrupted cortical outline by MTMGs (Fig. 2b,c,d,f,g,h,j,k and l). Based on the relationship between the hard tissue part of the MTMG and the IAC, level II was subclassified into three subcategories as follows: (IIa) no contact (Fig. 2f), (IIb) the hard tissue of the MTMG simply contacting the IAC (Fig. 2g) and (IIc) the hard tissue intersecting into the IAC (Fig. 2h).

**Figure 3:**
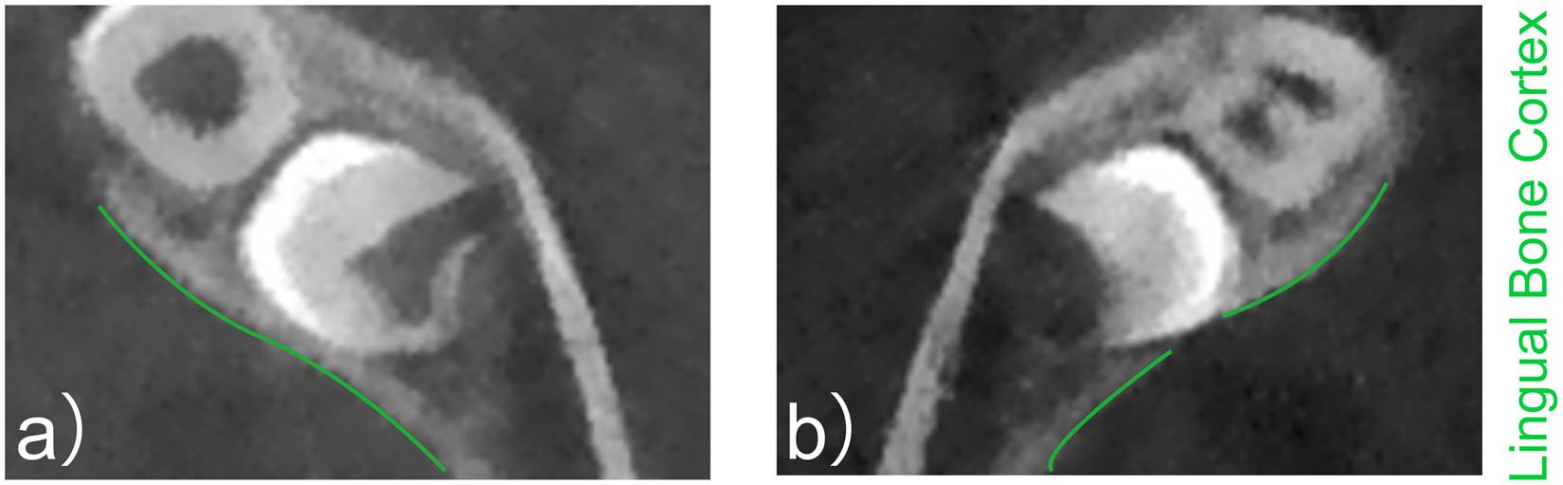
Observation of the cortical outline of the lingual bone cortex in the region of MTMGs. The green line represents the cortical outline. (**a**) Shows the continuous outline, (**b**) Indicates the disrupted outline.

The integrity of the lingual bone cortex was confirmed by the continuity of the lingual bone cortex. The axial images were observed from the top of MTMG to the bottom. The lingual bone cortex was presented as continuous white line in CBCT (Fig. 3a). The disrupted white line was defined as perforation (Fig. 3b).

Two observers measured all the data. Quantify interrater agreement between two observers was evaluated using kappa test by standard statistical software packages (SPSS, version 22.0, Chicago). The analysis was based on the data including the integrity of the lingual bone cortex and the relationship between IACs and MTMGs. A kappa value <0.40 was considered poor agreement, 0.40–0.75 was good agreement, 0.75–1.0 was excellent agreement.

### Variables and data analysis

Demographic variables were age and gender. All patients were divided into three age groups. Group A is composed of 12- and 13- year-old patients. Group B consists 14- and 15- year-old patients. Group C involves 16- and 17- year-old patients (Table 1). The development stages of the teeth were assessed according to Nolla calcification stage^10^ and classified as Nolla stage ≤6 or ≥7.

Data analysis was performed using the standard data package included with GraphPad Prism® 6.0 (GraphPad Software, Inc., La Jolla, CA, USA). Pearson chi-square test was used to compare the difference between each two age groups or the relationship between the variables and the enumeration data. The relationship among the three age groups was not analysed because it could only draw out unspecific result. *P*-value less than 0.05 was considered as significant.

## Result

The results of CBCT measurement were selected from one staff because the kappa value was approximately 0.8 between two staff chosen for the work. The discrepant results were finally negotiated by all authors to reach agreements.

### Continuity of cortical outline of IACs in the region of MTMGs in different groups

**Figure 4:**
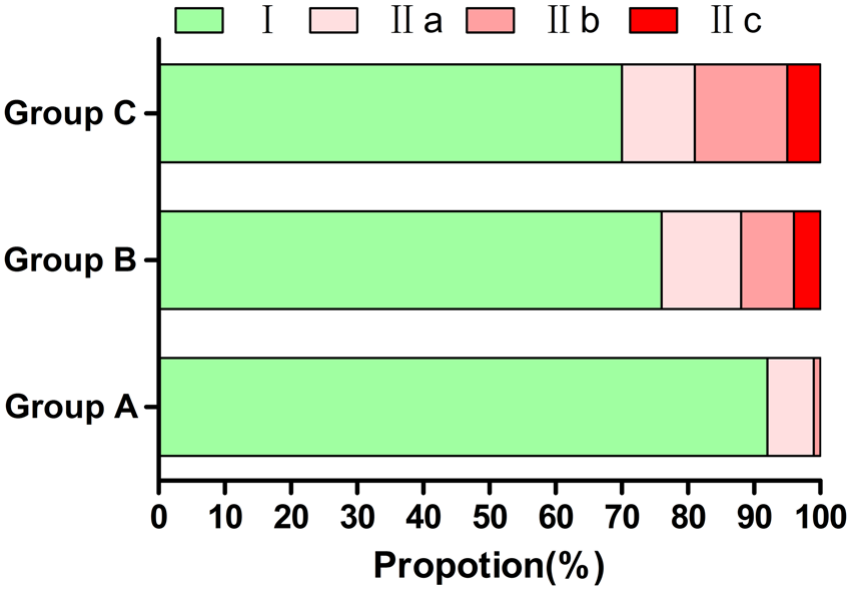
Information of relationship between MTMGs and IACs in different ages.

As shown in Table 2 and Fig. 4, the rates of disrupted cortical outline of IACs by MTMGs were 8%, 24% and 31%, respectively, in groups A, B and C. Meanwhile, group A showed a significantly lower incidence compared with group B (*P* = 0.002) or group C (*P* < 0.0001). No significant difference was observed between groups B and C (*P* = 0.2676). Males showed less rates than females (*P* = 0.0483), but no difference was found between the MTMG location. The proportions significantly increased after the initiated root formation (Nolla ≥ 7, *P < *0.0001) (Table 3).

**Table 2:** Information of relationship between MTMGs and IACs in different ages.

Group	age	I	II		
			IIa	IIb	IIc
A	12~13	92	7	1	0
B	14~15	76	12	8	4
C	16~17	70	11	14	5

**Table 3:** Continuity of the cortical outline of IACs in the MTMG regions in different groups.

Variable		Continuity of cortical outline		χ_2_
		Yes	No	
Gender	Male	120	22	*P* = 0.0483*
	Female	119	39	
Location	Left	120	31	*P* = 0.9322
	Right	119	30	
Development stage	Nolla ≤ 6	157	22	*P* < 0.0001****
	Nolla ≥ 7	82	39	
Age	Group A	92	8	*P* = 0.0020** (A vs. B)
	Group B	76	24	*P* = 0.2676 (B vs. C)
	Group C	69	31	*P* < 0.0001**** (A vs. C)

*P < 0.05; **P < 0.01; ***P < 0.001; ****P < 0.0001.

### Relationship between the hard tissue part of MTMGs and IACs in different groups

The rates of hard tissue part of MTMGs contacting IACs were 1%, 12% and 18%, respectively, in groups A, B and C. The cases of the hard tissue part of MTMGs contacting IACs in group A were significantly less than that of group B (*P* = 0.0016) or group C (*P* < 0.0001). Meanwhile, no significant difference was observed between groups B and C (*P* = 0.2348). In general, no significant relationship was found in different genders (*P* = 0.1865) or location (*P* = 0.3633) although the number of female was larger than that of male (20 *vs*. 11) and the number of the left side exceeded that of the right side (18 *vs*. 13). The incident rates significantly increased when the teeth in Nolla ≥ 7 stage (*P* < 0.0001) (Table 4).

**Table 4:** Comparison of different variables about the relationship between IACs and the hard tissue part of MTMGs.

Variable		IACs and MTMGs		χ_2_
		no contact	contact	
Gender	Male	131	11	*P* = 0.1865
	Female	138	20	
Location	Left	133	18	*P* = 0.3633
	Right	136	13	
Development stage	Nolla ≤ 6	171	8	*P* < 0.0001****
	Nolla ≥ 7	98	23	
Age	Group A	99	1	*P* = 0.0016** (A vs. B)
	Group B	88	12	*P* = 0.2348 (B vs. C)
	Group C	82	18	*P* < 0.0001**** (A vs. C)

*P < 0.05; **P < 0.01; ***P < 0.001; ****P < 0.0001.

### No significant differences in the rates of the lingual bone cortex perforation in different groups

With regard to perforation of the lingual cortex, the number of cases increased with age. However, no significant difference was found between ages, gender, location, stage of development and age. The rates of perforation of the lingual bone in groups A, B and C were 9%, 12% and 15%, respectively (Table 5).

**Table 5:** Comparison of different variables about perforation of the lingual cortex.

Variable		Perforation		χ^2^
		No	Yes	
Gender	Male	130	12	*P* = 0.0729
	Female	134	24	
Location	Left	132	19	*P* = 0.8594
	Right	132	17	
Development stage	Nolla ≤ 6	162	17	*P* = 0.1047
	Nolla ≥ 7	102	19	
Age	Group A	91	9	*P* = 0.4889 (A vs. B)
	Group B	88	12	*P* = 0.5348 (B vs. C)
	Group C	85	15	*P* = 0.1917 (A vs. C)

*P < 0.05; **P < 0.01; ***P < 0.001; ****P < 0.0001.

## Discussion

The tooth germ is an aggregation of cells that eventually forms a tooth in the dental lamina organised into three parts as follows: the enamel organ, the dental papilla and the dental sac or follicle^11^. In general, the germs can be simply classified into two parts, namely, hard tissue and soft tissue. Clinically, MTMG extraction is often performed from the age 12–13 years old when the MTMG is in the calcification level and the second permanent molar has emerged into oral cavity. The reasons of extraction are usually orthodontic treatment need or prophylactic extraction. However, some complications may occur during MTMG extractions, including inferior alveolar nerve (IAN) and lingual nerve injury. Therefore, the characteristics of adjacent anatomy were investigated in the present study through the CBCT images. In addition, these anatomical characteristics suggested that the risk of IAN and lingual nerve injury is lowest in the 12 to 13 age group during MTMGs removal.

IAN injury is a serious complication during teeth extraction. The anatomical factors for IAN injury were the location of MTMGs related to the IAC, the degree of impaction and the root development^12^. Among the relationship between MTMGs and IAC, the cortical integrity of IAC was an important factor in predicting post-operative IAN paraesthesia, and the degree of cortical interruption was positively correlated with the IAN injury^13^. The occurrence rate of IAN injury has been proved to be increased if the third molars intersect with IAC, especially when the molars were on the buccal side of IAC. Xu^14^ and his colleagues assumed that the IAN would be compressed by the adjacent tooth when elevator was inserted in the space between the buccal bone and tooth which may assert a force to the lingual side. During the MTMG removal, the IAN is impossible to be exposed to the wound in the level I categories because of IAC integrity. Therefore, IAC in level I can be defined as the ‘low risk of IAN exposure group’, whereas cases in level II were considered as the ‘high risk of the IAN exposure group’, which referred to the caution of curettage of tooth socket to prevent IAN injury. Furthermore, based on the possibility of nerve injuries and the relationship between the hard tissue part of the MTMG and the IAC, MTMGs in levels IIb and IIc were classified into ‘high risk of the IAN injury group’. Meanwhile, the rest of MTMGs (levels I and IIa) were regarded as ‘low risk of the IAN injury group’. If an MTMG was determined as high risk of the IAN injury, the indication of tooth extraction should be deliberated.

With regard to the lingual nerve injury, CBCT can only show the thickness of the lingual bone cortex and the lingual nerve cannot be demonstrated in the CBCT image. The risk of lingual nerve injury is often assessed through observation of the lingual bone cortex. The anatomical factors of lingual nerve damage were the perforation of the lingual cortex because the deficiency of the lingual bone in the retromolar area would provide lingual nerve vulnerability^15^. The perforation was found to be associated with the angulation of the teeth with mature roots^15^. However, all the enrolled teeth in the present study were tooth germs, which may be the reason for no significant difference between each two age groups. Furthermore, this condition must be given considerable attention to the cases with perforation of the lingual cortex.

CBCT can not only be accurate to predict IAN exposure versus panoramic^16^ but also can provide more information about the anatomic factors of nerve paraesthesia. CBCT can show multiple perspectives of IAN/MTMG anatomical location, the potential risk of exposure of lingual soft tissue (including the lingual nerve) with low-radiation dose. In this study, some data were provided to demonstrate the anatomical characteristics of MTMGs in ages of 12–17 years, which may provide some anatomical information to doctors. To the best of our knowledge, our study is the first to summarise the characteristics of adjacent anatomy of MTMGs. However, some limitations were also observed in our study. The patients in our work were all Chinese, and our data can only show some information of assessment of nerve injury from the standpoint of anatomy.

## Conclusion

According to the CBCT images, the possibilities of disrupted cortical outline of IACs by MTMGs or the hard tissue part of MTMGs contacting IACs were significantly lower in 12- or 13-years-old than that in 14- to 17- age groups. Meanwhile, no difference can be found in the perforation rate of lingual bone cortex in all 12 to 17- age groups. It suggested anatomical factors contributed the lest to the risk of inferior alveolar nerve and lingual nerve injury in the 12 to 13 age group during removing the MTMG removal.
